# Past and Future of Neurotrophic Growth Factors Therapies in ALS: From Single Neurotrophic Growth Factor to Stem Cells and Human Platelet Lysates

**DOI:** 10.3389/fneur.2019.00835

**Published:** 2019-08-02

**Authors:** Flore Gouel, Anne-Sophie Rolland, Jean-Christophe Devedjian, Thierry Burnouf, David Devos

**Affiliations:** ^1^Department of Medical Pharmacology, Lille University, INSERM UMRS_1171, University Hospital Center, LICEND COEN Center, Lille, France; ^2^Graduate Institute of Biomedical Materials and Tissue Engineering, College of Biomedical Engineering, Taipei Medical University, Taipei, Taiwan; ^3^International PhD Program in Biomedical Engineering, College of Biomedical Engineering, Taipei Medical University, Taipei, Taiwan; ^4^International PhD Program in Cell Therapy and Regeneration Medicine, College of Medicine, Taipei Medical University, Taipei, Taiwan; ^5^Department of Neurology, Lille University, INSERM UMRS_1171, University Hospital Center, LICEND COEN Center, Lille, France

**Keywords:** Amyotrophic lateral sclerosis, growth factors, therapeutic, stem cell, human platelet lysate

## Abstract

Amyotrophic lateral sclerosis (ALS) is a fatal neurodegenerative disease that typically results in death within 3–5 years after diagnosis. To date, there is no curative treatment and therefore an urgent unmet need of neuroprotective and/or neurorestorative treatments. Due to their spectrum of capacities in the central nervous system—e.g., development, plasticity, maintenance, neurogenesis—neurotrophic growth factors (NTF) have been exploited for therapeutic strategies in ALS for decades. In this review we present the initial strategy of using single NTF by different routes of administration to the use of stem cells transplantation to express a multiple NTFs-rich secretome to finally focus on a new biotherapy based on the human platelet lysates, the natural healing system containing a mix of pleitropic NTF and having immunomodulatory function. This review highlights that this latter treatment may be crucial to power the neuroprotection and/or neurorestoration therapy requested in this devastating disease.

**Graphical Abstract:**
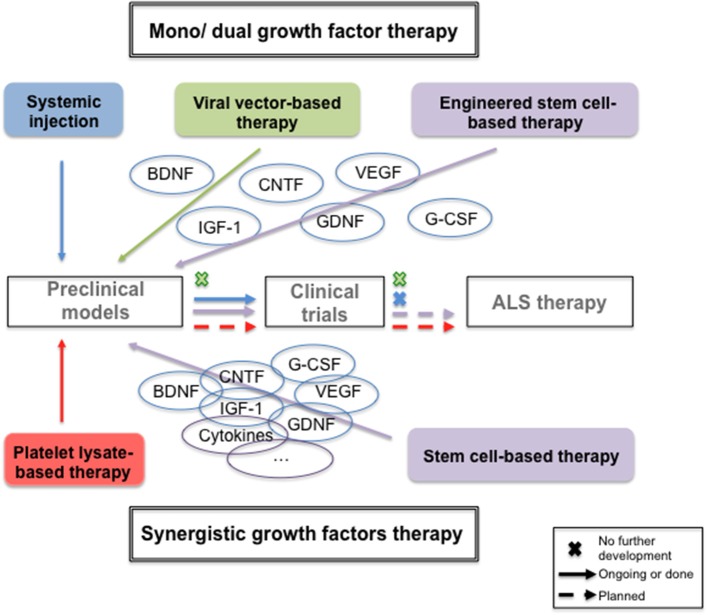
From single to synergistic neurotrophic growth factors therapies.

## Introduction

Amyotrophic lateral sclerosis (ALS) is a fatal neurodegenerative disease affecting the upper and lower motor neurons in the cerebral cortex, brainstem and spinal cord that lead to a progressive, irreversible muscle paralysis, and swallowing and respiratory dysfunctions. Death eventually occurs 3–5 years after diagnosis (1). The majority of ALS cases (90%) are sporadic with unknown cause (2). To date, there is no curative treatment in ALS. Therefore, the development of new and effective treatment is highly urgent. Among the different approaches, the delivery of neurotrophic factors (NTFs) is explored since the 90's because NTFs are necessary to regulate several physiological processes such as neuronal differentiation and survival, axonal outgrowth and synapses maintenance (3–5), proliferation and differentiation of stem cells in the nervous system (6–9). Therefore, these trophic factors represent a promising therapeutic strategy to treat neurodegenerative diseases (10) such as ALS.

## Preclinical Evidence of Neurotrophics Growth Factors Abilities to Treat Amyotrophic Lateral Sclerosis (Table 1)

### Recombinant NTFs Delivery by Injection

Some trophic factors have been demonstrated to promote cell survival and be protective in both *in vitro* and *in vivo* models of neuronal degeneration: Ciliary Neurotrophic Factor (CNTF), Brain-derived Neurotrophic Factor (BDNF), Glial-Derived Neurotrophic Factor (GDNF), Insulin-like Growth Factor 1 (IGF-1), Vascular Endothelial Growth Factor (VEGF), and Granulocyte-Colony Stimulating Factor (G-CSF). *In vivo* experiments performed in ALS models using single recombinant growth factors are described in this section.

**Table 1:** Different routes of NTFs delivery and therapies in pre-clinical models.

**NTF**	**Delivery route**	**Model**	**Outcomes**	**References**
**RECOMBINANT NEUROTROPHIC GROWTH FACTORS**				
CNTF	I.P	*pmn/pmn* mice (20–21 d)	MP+, S+	(11)
	S.C	Wobbler mice	MP+, MC+	(12–14)
BDNF	S.C	Wobbler mice	MP+	(14)
GDNF	S.C	*pmn/pmn* mice (15–18 d)	No effect	(15)
VEGF	I.P	SOD1^G93A^ mice (74 d)	MP+, DDO+, S+11 d	(16)
	I.C.V	SOD1^G93A/LSd^ rats (60 d)	MP+, DDO+, S+10 d	(17)
	I.S.P	Excitotoxic model in rats	MP+, DDO+, S+10.5 d, +5 d	(18, 19)
**Viral vector based gene therapy**				
**AAV-NTF**				
IGF-1	I.M	SOD1^G93A^ mice (90 d)	MP+, S+22 d	(20)
	I.S.P	SOD1^G93A^ mice (60 d)	MP+, DDO+, S+12.3 d ♂	(21)
	In D.C.N	SOD1^G93A^ mice (88–90 d)	MP+, S+14 d	(22)
	I.M	SOD1^G93A^ mice (60 and 90 d)	MP+, DDO+, S+29 d and +15 d ♂, +24 d and +14 d ♀	(23)
	I.V	SOD1^G93A^ mice (90 d)	MP+, S+10 d	(24)
	I.C.V	SOD1^G93A^ mice (80–90 d)	DDO+, S+12 d	(25)
VEGF	I.C.V	SOD1^G93A^ mice (80–90 d)	DDO+, S+9 d ♂, +20 d ♀	(25)
	I.T	SOD1^G93A^ mice (90 d)	DDO+, S+12 d	(26)
GDNF	I.M	SOD1^G93A^ mice (90 d)	MP+, DDO+, S+16.6 d	(27)
	I.V	SOD1^G93A^ rats (25 d)	MP +/–, S–	(28)
G-CSF	I.S.P	SOD1^G93A^ mice (70 d)	MP+, DDO+, S+	(29)
**Stem cell based therapy**				
**AAV-NTF**				
hSC-NSC	I.S.P	SOD1^G93A^ rats (56–62 d)	MP+, DDO+, S+11 d	(30, 31)
gm hNSC line (VEGF)	I.T	SOD1^G93A^ mice (70 d)	DDO+, S+12 d	(32)
hSC-NPC	I.S.P	SOD1^G93A^ mice (40 d)	MP+, S+5 d	(33)
gm hNPC (GDNF)	I.S.P	SOD1^G93A^ rats (~80 d) rats (~80 d)	MP–, S–	(34, 35)
	Cortex	SOD1^G93A^ rats (~80 d) macaques	DDO+, S+14 d	(36)
hBM-MSC	I.S.P	SOD1^(G93A)dl^ mice (28 w)	MP+	(37)
		SOD1^G93A^ mice	MP+	(38)
mBM-MSC	I.V	SOD1^G93A^ mice (90 d)	MP+, S+17.3 d	(39)
gm hBM-MSC (GDNF, VEGF, GNDF/IGF-1, BDNF)	I.M	SOD1^G93A^ rats (80 d)	MP+, S+28 d and +18 d for GDNF, + 13 d for VEGF, +28 d for GDNF/VEGF	(40, 41)
mBM	I.S.P and I.M	mdf/ocd mice (6 weeks)	MP+	(42, 43)
mASC	I.V	SOD1^G93A^ mice (76–77 d)	MP+, S–	(44)
hASC	I.V and I.C.V	SOD1^G93A^ mice (70 d)	MP+, DDO+, S+	(45)
hUCBC	I.V	SOD1^G93A^ mice (56 d, 66 d)	DDO+, S+21 d, +38.5 d, +23.8 d	(46–48)
		SOD1^G93A^ mice (60 and 90 d)	MP+, S+10 d	(49)
	I.T	SOD1^G93A^ mice	No effect	(50)
	I.S.P	SOD1^G93A^ mice (40 and 90 d)	MP+, S+6 d for 40 d mice	(51)
	I.C.V	SOD1^G93A^ (70 d) Wobbler mice (28 d)	MP+, S+18 d MP+	(52)
gm hUCBC (VEGF, GDNF, and/or NCAM)	I.V	SOD1^(G93A)dl^ mice	MP+, S+	(53, 54)

*I.P, intraperitoneal; I.M, intramuscular; I.V, intravenous; I.C.V, intracerebrovascular; I.S.P, intraspinal; I.T, intrathecal; S.C, subcutaneous; DCN, deep cerebellar nuclei; gm, genetically modified for expression of NTFs in brackets; hSC-NSC: human spinal cord-neural stem cell; m/hBM-MSC, murine/human bone marrow-mesenchymal stem cell; m/hASC, murine/human adipose derived MSC; hUCBC, human umbilical cord blood cells. Main results are summarized as follow: MP, motor performance; DDO, delay of disease onset; S, survival. The age of the model at the treatment is noted in brackets (d, days old; w, weeks). +, improvement; –, deterioration. ♂, male; ♀, female*.

CNTF, one of the first NTF studied in ALS models, injected intraperitonally in *pmn/pmn* mice, mouse model for human spinal motor neuron disease (11) or subcutaneously in wobbler mice (12) improved motor function and survival, and decreased neuronal degeneration and muscle atrophy (13). In addition, Mitsumoto et al. demonstrated a synergic effect of CNTF and BDNF, respectively, to arrest disease progression for 1 month (14).

The fusion protein BDNF with the c fragment of the tetanus toxin (BDNF-TTC) exhibited enhanced neuroprotective effect in SOD1^G93A^ ALS mice model, but no synergic effect was observed compared to TTC alone (55). Recently, motor function improvement and less neuronal loss were observed in SOD1^G93A^ mice treated with the flavonoid 7,8-dihydroxyflavone, a small-molecule mimicking the effect of BDNF (56). Two receptors binding the BDNF, p75^NTR^ and TrkB.T1, were highlighted in SOD1^G93A^: a decreased of p75^NTR^ expression correlated with a delay of mortality and motor impairment (57); a deletion of the TrkB.T1 increased survival and delayed motor deficit (58).

Treatment with encapsuled GDNF-secreting cells in *pmn/pmn* mice did not impact motor neuron degeneration and lifespan (15). The authors suggest a combined treatment for GDNF with others NTFs. Recently, astrocytic GDNF triggered by the tumor necrosis factor α (TNFα) was highlighted in the SOD1^G93A^ mice, and found to limit motor neuron degeneration and disease progression (59).

Intraperitoneal (16) or intracerebroventricular (17) injection of VEGF at doses of 1 g/kg/d and 0.2 μg/kg/d in SOD1^G93A^ mice and rats, respectively, increased lifespan and improved motor performance. Similar data were observed in a sporadic model of ALS rats induced by excitotoxic administration of AMPA (60, 61).

Finally, protective properties of G-CSF were observed in SOD1^G93A^ mice when delivered continuously at dose of 30 μg/kg/d (18). Indeed, disease progression was reduced and survival increased by rescuing motoneurons. Similar results were obtained with subcutaneous injection of pegfilgrastim, a more stable analog of G-CSF (19).

As protein infusion has known drawbacks (invasive method of delivery, protein stability over time, short half-life) others strategies, such as viral vector-based gene therapy and stem cell-based therapy have been developed to express NTFs of interest and avoid chronic injection.

### NTFs Delivery by Viral Vector-Based Gene Therapy

Many studies focused on IGF-1. The intramuscular injection of adeno-associated viral (AAV)-IGF-1 in SOD1^G93A^ mice before or at the time of disease symptoms delayed disease onset and increased lifespan (20). Intraparenchymal spinal cord delivery was also tested, showing higher expression of IGF-1 but partial rescue (21), whereas a stereotaxic injection into the deep cerebellar nuclei significantly extended mice lifespan (22). Recently the injection of self-complementary adeno-associated viral vector 9 (scAAV9), a more efficient transducing agent for IGF-1, extended survival, and motor performance of SOD1^G93A^ mice when injected either intramuscularly (23) or intravenously (24). Also, the intracerebroventricular injection of AAV4-VEGF was studied and gave similar results than AAV4-IGF-1 by slowing disease progression. No combined effect of these 2 constructions was observed in SOD1^G93A^ mice (25). Similarly the intrathecal injection of scAAV9-VEGF showed positive impact on lifespan and motor performance in mice (26). The AAV-GDNF, injected intramuscularly in SOD1^G93A^ allowed expression of the protein at the sites of injection, a retrograde transport in anterior horn neurons, and was associated with a delay in the onset and the progression of the disease (27). However, the systemic injection of AAV9-GDNF in SOD1^G93A^ rats showed limited functional improvement and no survival extension (28). Finally the efficacy of intraspinal delivery was showed for AAV-G-CSF in SOD1^G93A^ mice with minimal systemic effects (29).

### NTFs Delivery by Stem Cell-Based Therapy

Different types of stem cells exist—based on their source, clonogenic capacity, differentiation potential and availability—and exert a paracrine effect, suitable for therapy in neurodegenerative disease such the ALS (62–65). We mainly focus here on stem cells with potential clinical application, engineered or used as such, e.g., a mix of NTFs.

#### Neuroprotection With Neural Stem Cells (NSC) and Neural Progenitor Cells (NPC)

Human NSC graft into lumbar protuberance of SOD1^G93A^ rats was shown to delay the onset and the progression of the disease, with their integration into the spinal cord (30, 31). Similarly, the intraspinal administration of human NPC delayed the progression of the disease in SOD1^G93A^ mice (33).

NSC were also engineered to secrete specific one. Intrathecal transplantation of human NSC overexpressing VEGF in SOD1^G93A^ mice delayed the onset of the disease and increased survival with an integration and differentiation of NSC-VEGF into the spinal cord (32). Human neural progenitor cells NPC (hNPC) were also genetically modified to secrete GDNF. The transplantation of such engineered cells in SOD1 rats were integrated into the spinal cord, limited motoneuron degeneration but failed to improve motor function (34, 35). However, the transplantation of hPNC-GDNF into the cortex extended the survival of SOD1^G93A^ rats and was safe for primates (36).

#### Mesenchymal Stromal Cells (MSC)

Bone marrow (BM) MSC (BM-MSC), when injected intraspinally (37, 38) or intravenously (39) in SOD1^G93A^ mice, allowed decreased motoneurons degeneration, improved survival and motor function, prevented pro-inflammatory factors. Indeed, MSC display immunomodulatory properties by secreting anti-inflammatory cytokines such as TGF-β or IL-10 (66) Since neuroinflammatory markers were detected in neural tissues of ALS patients (67) promising results can be expected with MSC based therapy. Moreover, intramuscular transplantation of human BM-MSC genetically modified to secrete GDNF in SOD1^G93A^ rats, showed a decrease in motoneuron loss and an overall increased lifespan (40). In addition they demonstrated a synergic effect of the combined intramuscular delivery of hMSC-GDNF and hMSC-VEGF with an increased survival, protection of neuromuscular junction and motoneuron degeneration, greater than either growth factor delivered individually (41). Even though human BM-MSC injections have positive effects on the disease progression, it should be noted that the whole BM intraspinally transplanted showed a greater improvement of motor functions than BM-MSC in *mdf/ocd* mice (42) and increased motoneurons survival when intramuscularly transplanted (43).

Others reported positive results with adipose derived MSC when administrated by systemic (44), or intracerebroventricular administration (45).

#### Human Umbilical Cord Blood (hUCB)

The first study performed on SOD1^G93A^ mice irradiated and transplanted intravenously with hUBC mononuclear cells (MNC), showed a delay in the onset of symptoms and increased the survival (46, 47). Transplanted cells integrated regions of motoneuron degeneration and expressed neural markers (48). Recently, the efficiency of chronic intravenous injections of UCB MNC in symptomatic SOD1^G93A^ mice was demonstrated, with increased lifespan and reduced inflammatory effectors (49). Similarly, the intraspinal as the intracerebroventricular injection of hUCB in pre-symptomatic SOD1^G93A^ or wobbler mice increased survival and motor performance (51, 52). However, intrathecal administration of hUCB did not affect the lifespan of motor function of ALS mice (50).

Some authors engineered hUCB MNC to secrete some NTFs or to enhance homing at the site of degeneration (68, 69). Recently, transplanted hUCB transduced with AAV encoding VEGF, GDNF and/or neural cell adhesion molecule (NCAM), led to a high rate of SOD1^G93A^ mice survival and improved motor function. Moreover, transplanted cells were detected 1 month after grafting into the lumbar spinal cord (53, 54).

## Clinical Trials With Growth Factors: Evidence and Hypothesis for the Failure

Regarding the promising effects obtained in ALS animal models, clinical trials were conducted to examine the neuroprotective effects of these growth factors therapies in ALS patients (Table 2).

**Table 2:** Clinical trials with growth factors.

**NCT number**	**NTF**	**Delivery method**	**Phase and status of the trial**	**Cohort size**	**Outcomes**	**References**	**Year**
**PROTEIN INFUSION**							
Not provided	CNTF	SC	Phase I, terminated	57	No adverse neurologic effects, safe, and tolerated	(70)	1995
Not provided		SC	Phase I, terminated	570	No beneficial effect, adverse events dose related, increased number of death at the highest dose, no beneficial effect on ALS progression	(71)	1996
Not provided		SC	Phase II/III	730	Disease progression not modified, minor adverse side effects	(72)	1996
Not provided		IT	Phase I	4	Pain syndromes dose-related, no systemic side effect, no improvement, or worsen of motor function	(73)	1997
Not provided	BDNF	SC	Phase I/II, terminated	283	Tolerated, Trend of improved survival, less deterioration of predicted FVC	(74)	1995
Not provided	BDNF	SC	Phase III	1 135	Disease progression not modified, Patients with early respiratory impairment and with altered bowel function showed benefit	(75)	1999
Not provided	BDNF	IT	Phase I/II, terminated	25	Well tolerated, feasible	(76)	2000
Not provided	BDNF	IT	Phase III, terminated	17	No adverse events, no effect	(77)	2003
Not provided	BDNF	IT	Phase II/III, terminated	13	No effect	(78)	2005
Not provided	IGF-1	SC	Not specify	266	Slowed the progression of functional impairment, slow the decline in health-related quality of life	(79)	1997
Not provided		SC	Not specify	183	Safe and well-tolerated, no effect	(80)	1998
Not provided		IT	Not specify	9	No serious adverse effect, modest beneficial effect	(81)	2005
NCT00035815		SC	Phase III, completed	330	No benefit	(82)	2008
Not provided	G-CSF	SC	Phase I, terminated	13	Safe, less decline of ALSFRS score	(83)	2009
Not provided		SC	Phase I, terminated	39	Safe, no significative effect on ALSFRS score	(84)	2010
NCT00397423		Not specify	Phase II, completed	40	Not available		
NCT01999803	VEGF	ICV	Phase I, terminated	15	Not available		
NCT02269436		ICV	Phase I, terminated	11	Not available		
NCT01384162		ICV	Phase I/II, terminated	15	Not available		
**STEM CELLS**							
**NCT number**	**Type of stem cells**	**Delivery method**	**Phase and status of the trial**	**Cohort size**	**Results**	**References**	**Year**
NCT01348451	NSC	ISP	Phase I	12	No major adverse events	(85, 86)	2012
NCT01730716	NSC	ISP	Phase II, unknown status	18	Not available		
NCT02943850	NPC	ISP	Phase I/IIa, active, not recruiting	18	Not available		
NCT01640067	NSC	ISP	Phase I, completed	6	Safe approach, no increase of disease progression	(87)	2015
NCT00781872	MSC	IT, IV	Phase I/II, terminated	19	Safe and feasible, ALS-FRS score stable the first 6 months	(88)	2010
NCT03085706	PBMC	ISP	Phase NA, completed	14	Not available		
NCT01933321	HSC	IT	Phase II/III, completed	14	Not available		
NCT01609283	MSC	IT	Phase I, active, not recruiting	27	Not available		
NCT01142856	MSC	IT	Phase I, completed	1	Not available		
NCT00855400	MSC	ISP	Phase I/II completed	11	No severe adverse event, no acceleration in the rate of decline, possible neurotrophic activity	(89)	2012
NCT02286011	MC	IM	Phase I, active, not recruiting	20	Not available		
NCT00855400	MC	ISP	Phase I, completed	11	Safe approach, no worsening of the disease	(90)	2016
NCT03268603	MSC	IT	Phase II, recruiting	60	Not available		
NCT01254539	MSC	ISP, IT	Phase I/II, completed	63	Infusion of MSC produces spinal changes unrelated with clinical events and disease worsening	(91)	2013
NCT01363401	MSC	IT	Phase I/II, completed	64	Possible benefit lasting at least 6 months with safety	(92)	2018
NCT02917681	MSC	IT	Phase I/II, recruiting	28	Not available		
NCT02987413	MSC	IT	Phase I, completed	3	Not available		
NCT02290886	MSC	IV	Phase I/II, active, not recruiting	52	Not available		
NCT01051882	MSC	IM or IT	Phase I/II, completed	12	Safe and tolerated, no serious adverse event, possible benefits on ALS-FRS score, and percentage of FVC	(93)	2016
NCT01777646	MSC	IM + IT	Phase IIa, completed	14			
NCT03280056	MSC	IT	Phase III, Recruiting	200	Not available		
NCT02017912	MSC	IM, IT	Phase II, completed	48	Not available		
NCT01759797	MSC	IV	Phase I/II, completed	6	No adverse events, ALS-FRS score reduced, FVC percentage reduced	(94)	2019
NCT01771640	MSC	IT	Phase I, completed	8			

*FVC, force vital capacity; HSC, hematopoietic stem cells; I, intramuscular; ISP, intraspinal; IT, intrathecal; IV, intravenous; MC, mononuclear cell; MSC, mesenchymal stem cells; NPC, neuronal progenitor cells; NSC, neural stem cells; NTF, neurotrophic factor, PBMC, peripheral blood mononuclear cell; SC, subcutaneous*.

### Trials Involving NTFs Protein Systemic Injections

#### CNTF

In 90's the ALS CNTF Treatment study group published results obtained in phase I (70) and phase II/III (72) clinical trials where enrolled patients received subcutaneous administration of recombinant human CNTF (rHCNTF) at different doses, 15 or 30 μg/kg, three times a week for 9 months. The phase II/III randomized, placebo-controlled evaluated the safety, tolerability, and efficacy. No statistically difference between rHCNTF-treated patients and placebo-treated patients were observed and side effects were sufficiently severe to limit dosing in many patients. A second trial, same year, did not show any positive effect either (71).

One year later, Penn et al. published results of a phase I clinical trial with intrathecal pump delivery (73). The disease progression was not modified either but no systemic side effects were observed. Thus, intrathecal administration may be the preferred route of administration. To our knowledge, no further clinical study are under investigation.

#### BDNF

Due to a promising phase I/II clinical trial showing the safety and efficacy of subcutaneous administration of BDNF in 1995, a phase III was designed (74). Results failed to demonstrate an effect on survival but *post-hoc* analyses showed that those ALS patients with early respiratory impairment showed benefit (75). One year later a phase I trial showed the feasibility of intrathecal method of delivery (76) but two other trials conducted in 2003 and 2005 felt to detect any efficacy (77, 78).

#### IGF-1

In the late 90's, two clinical trials used IGF-1 at a dose of 0.1 mg/kg/d by subcutaneous delivery and found contradictory and opposite results (79, 80). In 2008, a phase III showed no benefit of this route of delivery in 2 years of trials (82). In a pilot study conducted in 2005, intrathecal administration had beneficial effect using high doses of IGF-1 (3 μg/kg every 2 weeks) but it was not placebo-controlled (81).

#### G-CSF

Ten years ago, two pilot clinical trials with subcutaneous G-CSF administration at a dose of 5 μg/kg/d reported a trend for slowing down the disease progression (84) and a delay in motor decline (83). A Phase II clinical trial is under investigation but results are not yet available.

#### VEGF

Three clinical trials assessed the safety, tolerability, and the possible motor function improvement as well as survival time of the intracerebroventricular administration of 4 μg/d VEGF. To our knowledge, no results are published.

#### 6- Failure Hypothesis

Most of the clinical trials based on direct protein administration gave disappointing outcomes in view of the promising preclinical results. Different hypotheses can be raised to explain those failures (70–84):

- The route of administration: subcutaneous injection seems less efficient than the intrathecal one
- The minimal ability of these growth factors to cross the blood brain barrier
- The dose: highest safe dose in humans can be lower than those determined in animals, as the clinical trial with CNTF demonstrated
- The treatment start time: in animals, treatment start before the onset of the disease whereas in humans the diagnosis is performed at later stage
- The need of synergic association of numerous neurotrophic factors

### Trials Involving Adeno-Associated Viral Gene Therapy

To our knowledge, there is no reported clinical trial using adeno-associated viral gene therapy despite promising results obtained with SOD1^G93A^ mice. AAV2 and AAV9 are vectors having the greatest potential, one specific for neuron tissue, one passing the blood brain barrier and exhibiting neuronal tropisms, respectively. One of the drawbacks of genes therapies for ALS can be the safety. Indeed to stop delivery will not be possible if serious adverse events occur during the treatment.

### Trials Involving Stem Cell Therapy

Twenty-two trials involving stem cells-based therapy are registered on ClinicalTrials.gov. Most of them use MSC from different origins and few have results available. This section is an overview of all the known clinical trials.

#### Neural Stem Cells

In 2012, two trials sponsored by Neuralstem used NSC by intraspinal injection. The phase I did not show any adverse events (85, 86), but the phase II has an unknown status on the ClinicalTrials.gov website.

Recently, published results of a phase I trial, proposing transplantation of human NSCs into the lumbar spinal cord, demonstrated the safety and reproducibility of this cell therapy. Moreover, because the brain tissue used was from natural miscarriages, ethical concerns may be eliminated (87). An ongoing clinical trial concern neuronal progenitor cells engineered to produce GDNF. This is a phase I/IIA trial, active but not recruiting. No results are available for now.

#### Blood Cells

Two clinical trials, one using autologous peripheral blood mononuclear cell for intraspinal transplantation and one in phase II/III using hematopoietic stem cells for intrathecal injection were conducted and completed but no results were reported to our knowledge. One trial using autologous bone marrow mononuclear cells (90) for intraspinal injection showed the safety of the procedure.

#### Mesenchymal Stromal Cells

Among 14 clinical trials using MSCs from diverse origin such as bone marrow, adipose tissue or engineered to secrete particular NTFs, through diverse types of delivery (intrathecal, intraspinal, intramuscular, intravenous, or intraventricular), 5 have no published results, 4 are ongoing, and 5 are completed with published results. All of them are listed in the Table 2 and the last 5 are detailed below and involved the use of the bone marrow derived MSCs.

In 2012, a phase I/II, using autologous bone marrow MSCs administered by intraspinal delivery, was conducted. No severe adverse event were observed, no acceleration of the disease progression noticed and an increase of the motoneurons in the treated segments compared with the untreated segments for patients who died for unrelated reasons to the procedure. Thus, this trial demonstrates the safety of intraspinal infusion of MSCs and suggests their neurotrophic activity (89). In 2013, a phase I/II confirmed the safety of BM-MSC infusion (91).

In 2016, two clinical trials in small groups of patients, phase I/II, used bone marrow MSCs engineered to secrete NTFs. Intramuscular transplantation for early ALS patients and intrathecal transplantation for progressive ALS patients were evaluated. They concluded that both route of administration are safe and provide indications of possible clinical benefits that need to be confirmed on a bigger cohort (93).

In 2018, a phase I/II trial was initiated to evaluate the safety and efficacy of these cells through intrathecal delivery. A possible benefit seems to last at least 6 months with apparent safety (92). A phase II is required to evaluate long-term efficacy and safety.

Finally, recent phase I/II trials showed safety and feasibility of intravenous and intrathecal transplantation of autologous bone marrow MSCs (94). Indeed, no adverse events were reported and the ALS-FRS score and the force vital capacity percentage were significantly reduced. Additional trials with bigger cohort are needed.

To conclude, stem cells-based therapy as a future therapy to treat ALS patients is premature due to the lack of results. As for the protein infusion, some questions need to be considered:

- The delivery method
- The timing of intervention
- The number of cells to transplant to obtain a therapeutic efficacy
- The capacity of transplanted cells to migrate to the area of interest and to mature in the hostile environment
- The evaluation of the long-term efficacy

Nevertheless, trophic factors remain essential for neuronal maintenance and survival and remain a promising candidate to treat ALS patients. Another source of those factors can be the natural healing system, namely the platelet lysate, and a continuous infusion into the brain by intracerebroventricular (ICV) injection can be a route of administration, avoiding the potential problem with the blood brain barrier crossing.

## How to Improve Growth Factors Therapeutics in ALS: a New Therapeutic Approach Based on the Human Platelet Lysate

The lack of clinical efficacy of single NTF infusion, despite a good diffusion, required increasing the dose to a point where they finally induced poor tolerance (i.e., μg). A single NTF was therefore unable to induce the complex set of signaling pathways required to promote efficient neuroprotection. Platelets constitute abundant, natural sources of physiological balanced mixtures of many growth factors [e.g., Platelet Derived Growth Factor (PDGF), VEGF, IGF-1, EGF, or TGFβ) (95) and are used to enhance wound healing and tissue repair (96). In addition, they express adhesion molecules, secret chemokines (97) giving thus neuroinflammatory property to the platelate lysate that could be of an additional interest in ALS therapy. Interestingly, it was demonstrated that ICV injection of human platelet lysates significantly reduced infarct volumes in rats with permanent middle cerebral artery occlusion, improved motor function and promoted endogenous neural stem cells proliferation (98). Similar results were obtained with platelet rich plasma in ischemic rats (99). Moreover, intranasal (IN) administration of platelet lysates was demonstrated to be neuroprotective in Alzheimer and Parkinson's disease animal models (100, 101). To pursue with the neuroprotective potential of platelets lysate in neurodegenerative diseases, we developed a heated low protein human purified platelet lysate (HPPL) preparation, compatible with ICV and IN intermittent administration, to deplete fibrinogen, avoid thrombogenic, and proteolytic activities. We demonstrated its neuroprotective effect in *in vitro* and *in vivo* model of Parkinson's disease and its anti-inflammatory properties (102). To extend the concept to ALS, HPPL was tested on a motoneuron-like model and strongly protected from apoptosis and oxidative stress (103). Higher neuroprotection was obtained with HPPL compare to single growth factor or combination of 4 (PDGF, BDNF, BFGF, VEGF) and involved specific signaling pathway such as Akt and MEK (103). These results give a real hope for neuroprotective therapy and need to be confirmed in *in vivo* ALS model with ICV or IN administration of HPPL.

## Author Contributions

FG and A-SR wrote the manuscript. J-CD, TB, and DD critically revised the manuscript. All authors read and approved the submitted version.

### Conflict of Interest Statement

The authors declare that the research was conducted in the absence of any commercial or financial relationships that could be construed as a potential conflict of interest.
